# The Blood-Labyrinth Barrier: Non-Invasive Delivery Strategies for Inner Ear Drug Delivery

**DOI:** 10.3390/pharmaceutics17040482

**Published:** 2025-04-07

**Authors:** Zhangyi Yi, Xiaoying Wang, Ge Yin, Yu Sun

**Affiliations:** 1 Department of Otorhinolaryngology, Union Hospital, Tongji Medical College, Huazhong University of Science and Technology, Wuhan 430022, China; m202476309@hust.edu.cn (Z.Y.);; 2 Institute of Otorhinolaryngology, Union Hospital, Tongji Medical College, Huazhong University of Science and Technology, Wuhan 430022, China; 3 Hubei Province Clinic Research Center for Deafness and Vertigo, Wuhan 430022, China

**Keywords:** blood-labyrinth barrier, cochlear, drug delivery, stria vascularis, hearing

## Abstract

The inner ear is a relatively isolated organ, protected by the blood-labyrinth barrier (BLB). This barrier creates a unique lymphatic fluid environment within the inner ear, maintaining a stable physiological state essential for the mechano-electrical transduction process in the inner ear hair cells while simultaneously restricting most drugs from entering the lymphatic fluid. Under pathological conditions, dysfunction of the stria vascularis and disruption in barrier structure can lead to temporary or permanent hearing impairment. This review describes the structure and function of the BLB, along with recent advancements in modeling and protective studies related to the BLB. The review emphasizes some newly developed non-invasive inner ear drug delivery strategies, including ultrasound therapy assisted by microbubbles, inner ear-targeting peptides, sound therapy, and the route of administration of the cerebrospinal fluid conduit. We argue that some intrinsic properties of the BLB can be strategically utilized for effective inner ear drug delivery.

## 1. Introduction

The blood-labyrinth barrier (BLB) is a selective permeability system that separates the blood circulation from the fluids of the inner ear, maintaining a distinct environment within the inner ear labyrinth [1,2,3,4,5].

The inner ear is located deeply within the temporal bone. Due to the fine anatomical structure, the BLB was discovered nearly a century later than the blood-brain barrier (BBB). The discovery of the BLB is thanks to the in-depth studies on inner ear physiology, especially the studies of vascular distribution within the inner ear. In the 1970s, while investigating the origin of perilymph in the cochlea, scientists observed that the chemical composition of inner ear lymphatic fluid differed significantly from both blood and cerebrospinal fluid (CSF) [6]. The composition of perilymph resembled blood ultrafiltrate rather than CSF, suggesting the existence of a special barrier, just like the BBB, that protects the microenvironment of the inner ear. In 1974, Klaus Jahnke, by observing the permeability of horseradish peroxidase (HRP) from the cochlear vascular endothelium to inner ear fluid, proposed the existence of a blood-perilymph barrier [7]. In 1980, this concept was further morphologically defined through freeze-fracture electron microscopy [1]. Further studies revealed a more complex blood-endolymph barrier, formed by the epithelial marginal cells of the stria vascularis (SV) and endothelial cells, sometimes referred to as the blood-strial barrier [8,9]. In 1988, S. K. Juhn coined the term blood-labyrinth barrier to encompass these barrier systems between inner ear fluids and blood, identifying the stria vascularis as the main structure executing the barrier functions [10].

BLB is crucial for maintaining the relative stability of the ion concentration and the endocochlear potential (EP) within the inner ear lymphatic fluid. The ion concentration gradient between the endolymph and perilymph and the EP provides the physiological basis for the mechano-electrical transduction process in cochlear hair cells, which is a fundamental process to hearing [11]. Therefore, BLB dysfunction may be closely related to hearing loss caused by various factors such as noise [12], aging [13], genetic mutations [14], and inner ear inflammation [15]. The BLB also presents a challenge to drug delivery and the artificial intervention of inner ear homeostasis by blocking most drugs and molecules from entering the inner ear [16]. The primary objective of this review is to provide a comprehensive overview of the BLB and its critical role in the pathological mechanisms underlying hearing loss. We specifically emphasize several recently developed innovative approaches that demonstrate potential for non-invasive BLB penetration through strategic utilization of its intrinsic biological properties.

## 2. Stria Vascularis and Blood-Labyrinth Barrier

The inner ear barrier systems, or the blood-labyrinth barriers, can be divided into five separate barriers: the blood-endolymph barrier, the blood-perilymph barrier, the cerebrospinal fluid–perilymph barrier, the middle ear–perilymph barrier, and the endolymph–perilymph barrier. Two semipermeable boundaries within the cochlear duct, the Reissner’s membrane and the basilar membrane, divide the cochlear duct into three distinct fluid-filled chambers, separating the inner ear lymphatic fluid into endolymph and perilymph, each with markedly different compositions. The inner ear barrier system can be thus subdivided into the blood-perilymph barrier, blood-endolymph barrier, and the endo–perilymph barrier based on the pathways substances take from the blood into the inner ear lymphatic fluid [10]. The blood-perilymph barrier regulates ion and nutrient exchange between blood and perilymph (high Na⁺), primarily in the cochlear vasculature, while the blood-endolymph barrier (via the stria vascularis) controls K⁺ and nutrient transport to endolymph (high K⁺), maintaining its unique electrochemical potential. The endo–perilymph barrier (e.g., Reissner’s membrane) physically separates these fluids, preserving their distinct ionic gradients critical for hair cell mechanotransduction and auditory signal generation.

Furthermore, anatomical continuity exists between the inner ear lymphatic system and cerebrospinal fluid (CSF) through the scala tympani’s connection to the subarachnoid space in the posterior cranial fossa, establishing the cochlear duct as a crucial conduit linking cochlear compartments with the CSF system [17,18].

The BLB regulates ion and nutrient exchange between blood and inner ear fluids via endothelial tight junctions in the stria vascularis. The cerebrospinal fluid–perilymph barrier maintains fluid balance between CSF and perilymph through the cochlear aqueduct. The middle ear–labyrinth barrier, formed by the round window membrane, selectively permits small molecules while blocking pathogens from the middle ear. Reissner’s membrane sustains the endolymph–perilymph ionic gradient via ion channels and aquaporins. The basilar membrane mechanically transmits sound vibrations to hair cells while separating cochlear fluid compartments [19].

Broadly speaking, the so-called blood-labyrinth barrier refers to the entire barrier system that separates the cochlear fluids from other fluids like blood and CSF [4,16,19,20], especially for those early studies in the field [1,8,9,21,22]. Other terms such as intra-ear fluid barrier, labyrinth membranous barriers, and membranous labyrinth barriers are also used to refer to the barrier systems in the inner ear.

It is important to understand that this term (blood-labyrinth barrier) broadly includes all anatomical barrier structures that separate cochlear (and vestibular) fluids from the blood, CSF, and tissue fluid. It does not specifically refer to any particular location or barrier, nor does it distinguish between different capillaries, tissues (such as the stria vascularis, neurons, and organ of Corti), and inner ear fluids (endolymph, perilymph, strial fluid) within the cochlea.

In some recent references, however, this term is specifically used to describe the intrastrial fluid–blood barrier (or blood-endolymph barrier) within the stria vascularis [23,24,25], which highlights the unique role of the stria vascularis in separating endolymph fluid from blood and generating EP.

In this review, the term “blood-labyrinth barrier” is used in a broader sense, as it encompasses a more comprehensive concept of separating the inner ear fluids from blood and CSF. We also emphasize the function of the SV, as it covers the intrastrial fluid–blood barrier and plays an important role in generating EP and maintaining cochlear ion homeostasis.

### 2.1. Cell Components of BLB

**Figure 1:**
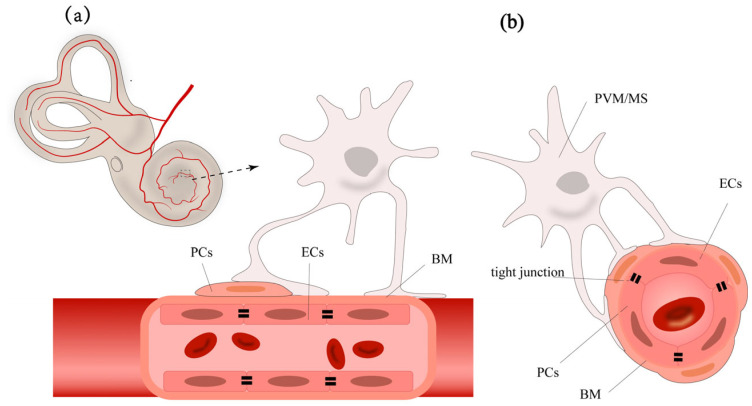
An illustration of the structure of blood-labyrinth barrier, showing the cross-section (**a**) and longitudinal section (**b**) of blood-labyrinth barrier. Endothelial cells (ECs) line the vessel lumen and are interconnected by tight junctions, forming the first layer of the blood-labyrinth barrier. These endothelial cells are surrounded by a dense basement membrane, which is shared with pericytes (PCs) that wrap around it. Perivascular resident macrophage-like melanocytes (PVM/Ms) extend their end-feet to cover a significant portion of the capillary surface, further enveloping the basement membrane.

The normal function of BLB relies on the integrity of the stria vascularis and the spiral ligament. The stria vascularis consists of marginal cells, intermediate cells, and basal cells (Figure 1). The capillary network traversing the intermediate cell layer includes endothelial cells (ECs), pericytes (PCs), and perivascular resident macrophage-like melanocytes (PVM/Ms). Each of these cells has unique functions, working together to form the barrier function of the stria vascularis. Understanding these cells’ roles is crucial for a comprehensive understanding of the BLB’s function.

The basic structure of the BLB is composed of the continuous ECs and their basement membrane (BM) of the capillaries in the stria vascularis and spiral ligament of the cochlear lateral wall, PCs, PVM/Ms, along with specific junctional structures between these cells [26]. This structure pattern is rather similar to the BBB (Table 1) and inner blood-retinal barrier (BRB) [27,28]. Endothelial cells connect through tight junctions, surrounded by the capillary basement membrane. PCs, around the capillaries, adhere to the surface of endothelial cells with their cell bodies and extend numerous processes wrapping around the capillaries, embedding into the basement membrane [29]. PVM/Ms are located in the intermediate cell layer, with dendritic cell bodies extending processes that attach to marginal cells on one side and to PCs and endothelial cells’ basement membrane on the other, regulating barrier integrity and permeability [30].

**Table 1:** Differences between blood-labyrinth barrier (BLB) and blood-brain barrier (BBB).

Feature	BLB	BBB
Anatomical location	Located in the stria vascularis of the cochlea and vestibular system	Found in brain capillaries, choroid plexus, and arachnoid epithelium
Primary function	Regulates solute exchange between blood and inner ear fluids (perilymph/endolymph) [31]	Protects the brain by restricting blood-borne substances and pathogens from entering the CNS [32,33]
Structural components	Endothelial cells, pericytes, and basement membrane	Endothelial cells, pericytes, astrocytes, and dual basement membranes (BM1 and BM2) [32]
Structural complexity	Simpler structure with sparsely distributed pericytes and no astrocytic end-feet	Complex neurovascular unit with astrocytes, pericytes, and neurons
Permeability	Allows passage of larger molecules (e.g., gentamicin); modulated by osmotic agents [8,19,34,35]	Highly selective for small lipophilic molecules; excludes most large molecules [27,36]
Pathophysiological role	Leakiness linked to ototoxicity, noise damage, and inflammation [21,37,38]	Dysfunction associated with neuroinflammation and neurodegenerative diseases [32]

### 2.2. Cells in Stria Vascularis

**Figure 2:**
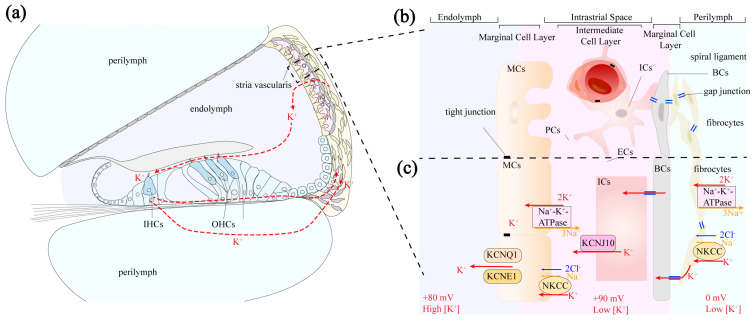
The structure of stria vascularis and K^+^ circulation: (**a**) The stria vascularis is located on the lateral wall of the scala media, as shown. The red dashed lines trace the circulation of K^+^ ions. K^+^ is expelled from the basal lateral side of hair cells and taken up by surrounding supporting cells. Through gap junctions, K^+^ is transported to root cells of the stria vascularis and released into the extracellular matrix; (**b**) the stria vascularis consists of three layers: a marginal cells (MCs) layer, intermediate cells (ICs) layer, and basal cells (BCs) layer. MCs and BCs are connected by tight junctions, while abundant gap junctions link intermediate cells, basal cells, and fibrocytes in the spiral ligament. The intermediate cell layer contains a dense capillary network that supplies nutrients to the stria vascularis. (**c**) A schematic view of ion-transport mechanisms in the generation of EP.

The stria vascularis is primarily composed of marginal cells (MCs), intermediate cells (ICs), and basal cells (BCs) [39] (Figure 2b). These cells are organized in layers, with the marginal cells closest to the endolymph, followed by the intermediate and basal cell layers. Tight junctions between MCs and BCs create a relatively closed fluid environment within the stria vascularis [15,40,41], known as the intrastrial space, containing intrastrial fluid. Intermediate cells, located between marginal and basal cells, extend numerous cell processes with complex folding structures interweaving, with capillaries traversing the intermediate cell layer [42].

Marginal cells directly face the cochlear scala media, with one side in contact with the endolymph and the other extending into the intrastrial space, where they send projections toward the ICs [43]. MCs are closely related to the unique high-K^+^ environment of the endolymph. The projections extending toward the intermediate cells are rich in mitochondria, providing the necessary energy for the active uptake of K^+^ from the intrastrial space via Na^+^-K^+^-ATPase and Na^+^-K^+^-2Cl^−^ cotransporters [44]. Lmx1a is essential for marginal cell maturation. Its loss abolishes KCNQ1 and BSND expression, preventing EP generation [45]. The marginal cells are connected by tight junctions on the side in contact with the endolymph, thereby separating the intrastrial space from the endolymph. Additionally, the active transcellular transport processes of marginal cells form the basis for selective substance entry into the endolymph.

The intermediate cell layer is located in the perivascular space between the marginal cell layer and the basal cell layer. This layer contains many components between the marginal cell layer and the basal cell layer, including capillary ECs, PCs, and PVM/Ms. The origin of ICs is rather complex, as both melanoblast- and Schwann cell precursor-derived ICs can be found during the embryonic stage [46]. The function of intermediate cells was believed to be the production of melanin granules [47] and participation in potassium ion transport [48]. Kcnj10 (critical for EP generation) expression decreases in intermediate cells, linking pendrin deficiency to EP loss [49]. Further studies reveal that these ICs exhibit characteristics of both macrophages and melanocytes. Therefore, the intermediate cells are also referred to as perivascular-resident macrophage-like melanocytes.

Basal cells are epithelial-like, elongated in shape, and connected to each other by tight junctions, forming a cohesive unit at the base of the stria vascularis, which separates the stria vascularis from the spiral ligament [41,50].

## 3. Stria Vascularis in K^+^ Circulation and EP Generation

As a selectively permeable physiological barrier, the BLB’s primary physiological significance lies in maintaining the relatively independent fluid environment within the membranous labyrinth, conducting ion transport to maintain normal cochlear EP (Figure 2), protecting cochlear cells such as hair cells from external damage, and ensuring normal physiological function.

The endolymph in the cochlear scala media exhibits a distinct ionic composition, with high potassium and low sodium levels, comparable to intracellular fluid. The high-potassium environment in the endolymph and the high EP between the endolymph and perilymph are essential for the mechanotransduction process in hair cells. It has been reported that the normal EP is dependent on transcription factor Lmx1a [45]. The potassium concentration in endolymph is approximately 140 mM, and the EP is about +80 to +100 mV, varying with experiment results and species [51,52].

When sound waves cause the basilar membrane to vibrate, stereocilia of hair cells bend, opening mechanically gated ion channels at the tips of the stereocilia, allowing potassium and calcium ions from the endolymph to enter the hair cells [53]. This potassium influx generates an electrical signal, initiating the conversion of mechanical sound waves into electrical nerve impulses transmitted to the brain. Subsequently, potassium ions exit the hair cells and enter the perilymph or are absorbed by supporting cells, which transport them to the stria vascularis in the lateral wall of the cochlea. Then, the potassium ions are reabsorbed and secreted back into the endolymph, forming the potassium ion cycle within the cochlea [54].

These ion channels and cell gap junctions work together to efficiently move potassium ions into the endolymph, maintaining its high potassium levels and EP. Additionally, the stria vascularis forms a barrier to prevent the entry of large molecules and potentially harmful substances from the blood into the endolymph, protecting the cochlear environment and ensuring normal hearing function.

### 3.1. Pathway of Potassium Ion Circulation

After K^+^ ions are expelled from the basal–lateral part of hair cells via the KCNQ4 potassium ion channel, they are taken up by surrounding supporting cells [54]. Through the epithelial cell gap junction system, K^+^ is transported to the root cells at the stria vascularis via gap junction proteins in Deiters cells, Hensen cells, and Claudius cells [44]. The root cells then secrete K^+^ into the extracellular matrix [55]. Potassium ions can also diffuse through the perilymph of the scala vestibuli and scala tympani to reach the spiral ligament. In the spiral ligament, some types of fibrocytes reabsorb K^+^ from the extracellular matrix and transfer it to type I fibrocytes [56]. Through gap junction proteins in the connective tissue cell gap junction system, K^+^ is transported across BCs to ICs and then secreted into the endolymph by MCs [44] (Figure 2a).

### 3.2. Generation of Endolymphatic Potential

The stria vascularis in the lateral wall of the cochlea plays a role in generating and regulating EP by secreting potassium ions from the perilymph into the endolymph [57]. The marginal cells of the stria vascularis are closely connected to the endolymph on one side and face the intermediate cells on the other. Tight junctions between marginal cells separate the stria vascularis lumen from the endolymph of the scala media. The apical membrane of the marginal cells, which faces the endolymph, contains K^+^ channels (KCNQ1 and KCNE1 channels). On the side facing the intermediate cells, the marginal cells extend numerous enlarged cellular protrusions into the layer of intermediate cells, which are rich in Na^+^-K^+^-ATPase and Na^+^-K^+^-2Cl^−^ cotransporters (NKCCs) to absorb K^+^ ions from the intrastrial space [52]. The intermediate cells transfer K^+^ into the intrastrial space via the Kir4.1 channel (an inward-rectifying potassium ion channel encoded by the KCNJ10 gene, also known as the KCNJ10 channel) [11].

Notably, Lmx1a is essential for marginal cell differentiation and stria vascularis formation, as its absence leads to loss of key proteins (BSND, KCNQ1, CD44) and failure of intermediate cell migration, resulting in EP abolition [45]. Single-cell RNA sequencing reveals pendrin (SLC26A4) expression in spindle cells maintains endolymph pH homeostasis, with its deficiency causing pH acidification and dysregulation of exosome-related genes like Annexin A1, impairing cellular communication [49].

The entire process of endolymphatic potential generation is as follows [44,58]: A significant influx of potassium ions through the Kir4.1 channel on the membrane of intermediate cells flows into the intrastrial space. Marginal cells actively uptake K^+^ from the intrastrial space through Na^+^-K^+^-ATPase and NKCCs then release the cytoplasmic potassium into the scala media via the apical KCNQ1 and KCNE1 channels. The Kir4.1 channel in intermediate cells plays a key role in EP formation, while marginal cells contribute indirectly (Figure 2c).

## 4. Selective Permeability of the Blood-Labyrinth Barrier

The cochlear fluid consists of two parts: endolymph and perilymph, which differ in their permeability to specific substances. In fact, the researches on the permeability of specific substances from blood to the cochlear fluid are often incomprehensive. In addition, many studies focusing on the permeability of the BLB only test the concentration of substances in perilymph [7,8,9]. Using the tracer ion trimethylphenylammonium (TMPA) (181 g/mol) as an indicator in perilymph, the BLB shows lower permeability compared to the BBB [2]. The selective permeability of the blood barrier in the stria vascularis is determined by the specific structure and function of capillary ECs, PCs, PVM/MS, and the base membrane [26].

The BLB exhibits inverse permeability to molecular size, with molecules < 100 Da crossing more readily [59]. Osmotic agents such as glycerol (92 Da) and urea (60 Da) can pass through the barrier and enter perilymph, while mannitol (182 Da) cannot [8]. While glycerol and urea exhibit partial lipid solubility, enhancing their diffusion, mannitol’s high polarity further limits membrane permeability. Even after extensive noise exposure, the mannitol concentration shows no difference compared to the control group [3].

Fluorescent tracers with relatively large molecular mass, such as cadaverine Alexa Fluor-555 (950 Da), BSA-Alexa Fluor-555 (66 kDa), and IgG Alexa 568 (200 kDa) can hardly penetrate through the BLB, but after disruption of PVM/Ms, they can accumulate in the lateral wall of the cochlea [30].

Ototoxic substances such as kanamycin enter the cochlear lymph fluid very slowly, with peak concentrations much lower than those in serum, yet they accumulate in specific cochlear cells and exert ototoxic effects [9,60]. Furosemide (330 Da) can also enter perilymph, with a relatively constant concentration before full recovery [8]. Similarly, after intravenous injection of ^3^H-marked taurine (125 Da), it can be detected in perilymph 1 and 2 h later [61].

Additionally, anionic sites have been reported on the BLB that act as an electrical charge barrier [62], which is associated with the selective transport of macromolecules. Hence, the cationic tracer polyethyleneimine (PEI) has been used to test the integrity of this charge barrier [62,63,64]. Ototoxic substances such as cisplatin and aminoglycosides are positively charged, which could combine with anionic sites and cause adverse effects.

As a result of the BLB, systemic drug administration often fails to penetrate the inner ear or exhibits significant delays due to the selective permeability of the BLB, leading to low drug concentrations in the inner ear while causing significant off-target effects elsewhere.

The permeability of the BLB varies under various conditions, including inflammation, noise exposure, and the disruption of BLB components. However, existing research on permeability is quite limited. Understanding the nature of BLB permeability under different circumstances may contribute to the discovery of suitable drugs that can pass through the BLB.

## 5. Inner Ear as a Former Assumed Immune Privileged Organ

The inner ear was once considered as one of the immune privileged organs since the presence of the BLB is similar to the BBB and BRB, which could restrict the entry of blood-borne immune effector cells and molecules [65], thereby theoretically reducing immune-mediated damage. The rather low immunoglobulin titer (1/1000 in serum) in perilymph seemed to support this assumption [66,67]. However, researchers have found that various stimuli, such as inflammation, noise exposure, and aminoglycoside drugs, can lead to widespread infiltration of lymphocytes and macrophages into the inner ear [68].

Compared to brain tissue, the inner ear is more sensitive in its response to antigens. When the antigen is placed into the inner ear of animals that have been pre-sensitized via systemic routes, the resulting immune response leads to an increased perilymphatic antibody titer, local antibody production, as well as lymphocyte infiltration and cochlear inflammatory damage [66,69]. Further studies reveal that macrophages in cochlear and PVM/Ms cells in the stria vascularis function as antigen-presenting cells and are associated with cochlear inflammatory responses [70]. The distribution of cochlear macrophages is reported to be approximately 62% in neural tissues and 36% in the spiral limbus and spiral ligament [71]. Following noise exposure, there is a marked increase in CD45+ cells, which are derived from the monocyte/macrophage lineage [72]. These cells accumulate in the spiral ligament and spiral limbus and perform a phagocytic function in the cochlea. PVM/Ms cells in the stria vascularis, on the other hand, regulate barrier permeability when exposed to noise [24]. Besides, immune cells (primarily monocytes) in circulation can enter the cochlea and infiltrate when exposed to acute damage [73]. Therefore, the significant immune response of the cochlea to external stimuli has led to the reconsideration of the inner ear as an immune-privileged organ.

However, immune cells are rarely reported in the organ of Corti, even after acoustic trauma and inflammation stimulation [71,72,74]. Even phagocytic cells found in the Corti region appeared only later after noise exposure (on the fifth day) and were located in the tunnel of Corti and the region of the outer hair cells, without causing damage to the cell structure of inner hair cells and supporting cells [75]. It is reasonable to assume that the organ of Corti, the most delicately organized part of the inner ear, is endowed with immune privilege. The main function of the organ of Corti is to convert mechanical stimuli into detectable action potentials through mechanotransduction channels, which is a core process in sound perception [76]. Immune cell infiltration, if it occurs, could disrupt the delicate microenvironment and compromise the functional integrity of sensory cells. The lack of a vascular system and the presence of a connective tissue cell gap junction system may also serve as protective factors against potential disruption of the endolymph microenvironment.

## 6. Pathological Mechanisms of Blood-Labyrinth Barrier Dysfunction

Many pathogenic factors can disrupt the function of the BLB, leading to sensorineural hearing loss (Table 2). The resulting types of hearing impairment include, but are not limited to, drug-induced hearing loss [77], noise-induced hearing loss [78], genetic hearing loss [79], and inner ear immune inflammation [70]. It is important to note that these pathological factors do not solely cause damage to the BLB; in fact, damage to the BLB may account for only a small portion of the causes of hearing loss. Drug-induced hearing loss ototoxic drugs, like other drugs, are limited in lymphatic fluid because of the BLB, and their peak concentration and time to peak in the inner ear labyrinth are typically lower than those in serum [9]. However, ototoxic drugs can enter the labyrinth and damage inner ear cells, particularly hair cells, through various pathways, causing ototoxic effects. Long-term, high-dose administration can also lead to drug accumulation, which surpass the toxic levels. Some drugs like loop diuretics can further exacerbate the ototoxicity of other drugs when used in combination.

**Table 2:** The pathological mechanisms of blood-labyrinth barrier dysfunction.

Pathological Factor	Mechanism of BLB Damage	Pathological Changes	Functional Consequences	Hearing Impact
Loop diuretics (e.g., furosemide) [80,81,82]	Block Na^+^-K^+^-2Cl^−^ cotransporter in marginal cells [80]	Stria vascularis edema;	Disrupted K^+^ recycling; temporary loss of EP [80]	Reversible hearing loss
		hypoxia-induced EC swelling [80];		
		transient EP disappearance [80]		
Cisplatin [34,64,83,84,85,86,87]	ROS generation [88];	ZO-1/cx26/cx43 downregulation [34];	Increased paracellular permeability	Permanent SNHL
	apoptosis [89]	TNF-α upregulation in SV [84]; mitochondrial dysfunction [88]	EP reduction (≥20 mV)	
Aminoglycosides (e.g., gentamicin) [90,91,92,93,94,95]	Accumulation in marginal cells [90];	Marginal cell lipid deposition [95];	Impaired K^+^ secretion	Progressive permanent damage
		ER swelling in intermediate cells [95];		
	TRPA1-mediated entry into hair cells [90,96]	lysosome formation [95]		
Genetic mutations [14,45,97,98,99]	Disrupted gap junctions in basal/intermediate cells [99]; developmental defect [45];	Impaired intercellular communication [99]; lack of key proteins [45];	Collapsed EP	Congenital/progressive SNHL
	ion channel defect [97,98,100]	ion channel dysfunction [97,98,100]		
Acoustic trauma [24,74,101,102,103]	Noise-induced ROS burst [103]; mechanical stress on SV capillaries [102]	HIF-1α/VEGF upregulation [101,102]; PC migration [101]; tight junction degradation [24,101,102]	Reduced capillary density; strial atrophy	Temporary/permanent threshold shift
Inflammatory responses [37,38,70,104,105,106,107]	Cytokine storm (TNF-α/IL-6) [70]; LPS activation of PVM/Ms [104,107,108]	Occludin/ZO-1/VE-cadherin downregulation [70]; macrophage infiltration [108]	Immunoglobulin leakage; antigen-specific immune attack on BLB	Immune-mediated SNHL

### 6.1. Loop Diuretics

Loop diuretics, such as furosemide and ethacrynic acid, are typically used to target the thick ascending limb of the loop of Henle in the kidney [82]. By blocking the Na^+^-K^+^-2Cl^−^ cotransporter, they inhibit the reabsorption of Na^+^, K^+^, and Cl^−^, rapidly increasing urine output. The marginal cells of the mammalian stria vascularis also express the Na^+^-K^+^-2Cl^−^ cotransporter, which is considered the target for loop diuretics causing ototoxicity [80]. Injection of ethacrynic acid can induce significant microcirculatory disturbances in the cochlear lateral wall, including stria vascularis edema, thickening, and cystic changes, potentially leading to the loss of the Na^+^ and K^+^ concentration gradient between the endolymph and perilymph, resulting in the disappearance of EP and temporary hearing loss [80]. These drugs temporarily block blood flow to the lateral wall, leading to hypoxia in capillary ECs, resulting in pathological changes such as stria vascularis edema, enlargement of intercellular spaces, and significant reductions in endolymphatic potential. Potent loop diuretics can lead to temporary hearing loss, which usually recovers over time without causing permanent hearing damage.

### 6.2. Cisplatin

Cisplatin-induced cochlear damage primarily affects hair cells but also directly impairs the function of stria vascularis cells, leading to dysfunction and a decrease in endolymphatic potential [86]. The ototoxic mechanism of cisplatin involves the production of reactive oxygen species, activation of intrinsic apoptotic pathways, mitochondrial dysfunction, and involvement of inflammatory cytokines [83]. In rats treated with cisplatin, strong expression of tumor necrosis factor-alpha (TNF-α) is observed in the cochlear spiral ligament and stria vascularis [85]. Cisplatin activates potassium channels in marginal cells of the stria vascularis, leading to the efflux of intracellular potassium ions, altering ion concentration and osmotic pressure within cells, which in turn activates apoptotic enzymes, inducing cell death. Hydration of cisplatin can neutralize the negative charge barrier formed by certain proteins on the basement membrane of capillaries, allowing negatively charged substances to cross the ECs more easily [86]. Cisplatin also reduces ZO-1, cx26, and cx43 expression on stria vascularis cells and leads to damage to the BLB’s charge barrier, resulting in the reduction in EP [84]. Cisplatin can also damage the BLB’s PCs by reducing viability and increasing toxic effects, which can be mitigated by dexamethasone and enhanced proliferation with PDGF-BB treatment [34].

### 6.3. Aminoglycoside Antibiotics

Aminoglycoside antibiotics are a series of classic ototoxic drugs; these antibiotics primarily cause damage to the cochlea and/or vestibular system [94]. Research on the direct effects of aminoglycoside antibiotics on the BLB is still insufficient. In mice subjected to prolonged injections of gentamicin, thinning of the stria vascularis, the presence of lipid structures within marginal cells, nuclear deformation, and swelling of the endoplasmic reticulum in intermediate cells have been observed, along with the formation of lysosome-like bodies [95]. Aminoglycosides may reach the cochlea by passing through capillaries to the marginal cells of the stria vascularis, then into the lymphatic fluid, and finally being absorbed by hair cells [90]. Fluorescently tagged gentamicin has shown that the concentration of gentamicin is higher in marginal cells than in basal and intermediate cells. It is generally believed that the primary target cells for aminoglycoside-induced ototoxic damage are the outer hair cells of the cochlea, with damage progressing from the base to the apex of the cochlea [94]. The mechanical transduction channels at the apex of hair cells, such as transient receptor potential channels (TRPA1), are considered pathways through which gentamicin enters the hair cells [93].

Due to the presence of the BLB, aminoglycoside antibiotics reach peak concentrations in inner ear fluids much more slowly than those in the blood, leading to two completely different outcomes regarding the combined use of aminoglycoside antibiotics and loop diuretics. When gentamicin concentration in the blood is higher than in the lymphatic fluid, using ethacrynic acid to disrupt the BLB can accelerate the entry of ototoxic drugs into the cochlea, rapidly reaching concentrations that damage hair cells [109]. Conversely, when the gentamicin concentration in the blood is lower than in the lymphatic fluid, using ethacrynic acid to disrupt the BLB helps promote the excretion of ototoxic drugs from the inner ear fluid, thereby reducing their continuous damage to hair cells [109].

In addition to the drugs mentioned above, animal experiments have shown that chronic salicylate poisoning can lead to a decrease of blood flow in the stria vascularis [81]; large doses of quinine can cause atrophy and degeneration of the stria vascularis [110]. These findings suggest that various drugs can accumulate in the inner ear and damage the stria vascularis, thereby affecting hearing health. Understanding the underlying mechanisms may help develop protective strategies, making the use of these ototoxic drugs safer.

### 6.4. Genetic Mutations

Genetic mutations affecting proteins involved in the BLB can lead to inherited forms of hearing loss. For example, mutations in genes encoding tight junction proteins [14,111], ion channels [11,100], or other structural components of the BLB can result in a compromised barrier function, including Norrie disease [112], Alport syndrome [113] and mutant of Light (Blt) [114], white spotting (Ws) locus [115], and estrogen-related receptor β (NR3B2) [116]. Importantly, knockout of Lmx1a disrupts stria vascularis formation by abolishing marginal cell differentiation (BSND and KCNQ1 loss) and intermediate cell recruitment (CD44 disappearance), leading to complete elimination of the EP and profound hearing loss [45]. The disruption of the BLB may allow the passage of potentially harmful substances from blood into the inner ear fluids, leading to progressive hearing loss. Mutations in genes encoding gap junction and ion transport proteins associated with the stria vascularis in the cochlea can weaken or impair BLB function. The gap junction proteins present in the cochlea include connexin 26 (encoded by the GJB2 gene) [14], connexin 30 (encoded by the GJB6 gene) [111], and connexin 43 (encoded by the GJA1 gene) [99]. These proteins are prominently expressed in the basal and intermediate cells of the stria vascularis; therefore, stria vascularis dysfunction is one of the mechanisms leading to genetic hearing loss caused [117]. Mutations of the ion channel proteins KCNQ1 and KCNE1 in marginal cells can cause Jervell and Lange–Nielsen syndrome (JLNS) related deafness, accompanied by severe arrhythmias [118]. Mice with Kcnq1 or Kcne1 deletions or spontaneous Kcel1 mutations exhibit significant hearing loss [119].

### 6.5. Acoustic Trauma

The mechanisms by which acoustic trauma alters BLB permeability are rather complex. Studies have shown that noise exposure can induce microcirculatory changes in the inner ear, including vasoconstriction, reduced blood flow velocity, increased blood viscosity, reduced local blood perfusion, capillary endothelial cell swelling, and increased permeability [21,120,121]. Additionally, noise exposure leads to structural and quantitative changes in stria vascularis cells, accompanied by upregulation or downregulation of various cytokines, decreased expression of junction proteins, and reduced energy metabolism capacity [14,24,102,122]. After noise exposure, hypoxia-inducible factor-1α (HIF-1α) and vascular endothelial growth factor (VEGF) are upregulated, inducing pericyte proliferation in the stria vascularis [103]. The downregulation of PEDF secretion by PVM/Ms decreases the synthesis of tight junction-related proteins (ZO-1, VE-cadherin) between ECs [24,30]. Furthermore, the expression of matrix metalloproteinases (MMP)-2 and -9 plays a role in regulating tight junction proteins, thereby influencing the integrity and permeability of the BLB [123]. Prolonged noise exposure can also lead to the shrinking of the stria vascularis, degeneration of spiral ligament fibrocytes, and a reduction in EP [124]. Noise exposure damages both the structure and cells of the cochlea, particularly affecting inner and outer hair cells. Compared to noise-induced decreases in endolymphatic potential, the direct damage to hair cells caused by noise exposure has a more immediate and significant impact on hearing loss.

### 6.6. Inflammatory Responses

Inflammatory responses can significantly impact BLB function. Inflammation reaction in the inner ear can be triggered by infections, autoimmune reactions, or acoustic trauma [70,74,104]. This leads to the release of inflammatory cytokines and other mediators that can compromise the integrity of the BLB. Increased permeability allows immune cells and other potentially harmful substances to infiltrate the inner ear fluids, leading to damage of cochlear cells and subsequent hearing loss [125].

The specific mechanisms by which inflammation increases BLB permeability are not yet fully understood. Generally, inflammatory factors can directly damage the vascular endothelium, leading to increased permeability and acting as inflammatory mediators to further promote the inflammatory response. Animal experiments have shown that directly injecting inflammatory factors and LPS into the inner ear through the round window membrane increases BLB permeability and disrupts the balance of the endolymph [104]. After injecting LPS into the middle ear, the permeability of blood vessels significantly increased, along with the activation of PVMs [38]. LPS-induced otitis media can lead to downregulation of connexin26 expression in the spiral ligament, thereby affecting the permeability of the inner ear BLB [126]. However, in inflammation induced by intraperitoneal injection of LPS in mice, despite the accumulation of macrophages in the spiral ligament, no decrease in EP or hearing loss was observed [105]. Additionally, during viral or bacterial infections, the increase in serum antiphospholipid antibodies may also lead to BLB dysfunction [127].

## 7. In Vivo and In Vitro Models for Studying the Blood-Labyrinth Barrier

### 7.1. In Vivo Models

Animal models are indispensable for studying the BLB and its role in inner ear function. Rodents, particularly mice and rats, are commonly employed due to their accessibility and relative physiological similarities to the human inner ear. Furthermore, they provide a valuable platform for testing potential therapeutic interventions, facilitating a deeper understanding of the physiological and pathological mechanisms underlying the BLB. A novel research have developed a minimally invasive ultrathin cochlear vascular window technique in mouse models, enabling real-time observation of blood flow changes in the inner ear [128]. In the inflammation process, PVM/Ms help maintain barrier integrity and trigger local inflammation after injecting bacterial LPS into the middle ear [108]. Further in vivo studies revealed that LPS disrupts the BLB by reducing the levels of occludin, ZO-1, and VE-cadherin between ECs, while simultaneously elevating the expression of MMP9 [38,104]. In the aging process, the structure and cell component of the BLB changes, with decreased capillary density and reduced numbers of PCs and PVM/Ms [129]. Acoustic trauma decreases capillary density and increases matrix protein deposition around PCs, with a phenotypic change in some PCs from negative to positive for α-smooth muscle actin [101]. This may explain the phenomenon of PCs migration following noise exposure, which is associated with the upregulation of platelet-derived growth factor beta (PDGF-BB) [130].

### 7.2. In Vitro Models

In vitro models often use specific cell types, typically ECs, to mimic the functions of barrier systems [131]. Various cells including ECs, PCs, and PVM/Ms that make up the BLB can be isolated and cultured in vitro to create culture-based two-dimensional (2D) specimens and investigate interaction between different cells [132]. In the LPS-induced in vitro infection model, the morphology and function of PCs and PVM/Ms change, thereby affecting barrier integrity [104]. In a transwell co-culture system with ECs, PCs, and/or PVM/Ms, Nhe6-knockout BLB-derived cell monolayers exhibited reduced electrical resistance and increased permeability [133].

The new culture platforms, such as organoids, microfluidic systems, and organ-on-a-chip technologies, have been developed to better mimic the three-dimensional environment of the body. This is particularly important in the study of the neurovascular unit due to the complex interaction between neurons, glial cells, and vascular cells [134]. The research on the BLB also benefits from such development. PCs and PVM/Ms are shown to be vital for supporting the integrity and organization of cochlear blood vessels by using three-dimensional culture models [129]. The human BLB on a chip model is developed based on PCs and ECs isolated from post-mortem human tissue to mimic the integrity and permeability of the BLB [135]. Using this model, inflammatory factors such as TNF-α, IL-6, and LPS are tested to demonstrate their disruptive effects on the barrier. By reducing the expression levels of ZO-1 and OCL, TNF-α is confirmed to have the most significant disruptive effect on the endothelial monolayer [37].

## 8. Current Inner Ear Drug Delivery Strategies

The presence of the BLB poses a challenge for the pharmacological treatment of inner ear diseases. Due to the barrier function of the BLB, systemic administration of drugs often results in limited effectiveness for inner ear conditions [136], with studies showing that only ~0.000005% of methylprednisolone crosses from the bloodstream to inner ear fluids [137]. Even when drug concentrations in the bloodstream are high following systemic administration, the concentration within the target organs of the inner ear remains low, as evidenced by human studies where dexamethasone concentrations in perilymph after systemic administration were 88-fold lower than those achieved through intratympanic injection [138]. To achieve effective therapeutic concentrations in inner ear tissues, clinicians have to increase the systemic drug dosage, which can lead to off-target effects and adverse reactions. Finding efficient, safe, and non-invasive (or minimally invasive) drug delivery methods is a key focus in otological research [16].

To overcome the barrier function of the BLB that limits the effectiveness of systemic treatments, traditional approaches often rely on local drug delivery methods, such as intratympanic or intracochlear administration [136]. Intratympanic injection delivers drugs through the eardrum into the tympanic cavity, allowing them to diffuse through the round window membrane into the inner ear’s lymphatic fluid. This method has been used for over half a century in the treatment of Meniere’s syndrome [139]. Although this method bypasses the BLB, it requires good contact between the drug and the round window membrane, and controlling the dosage is challenging due to variable RWM permeability and rapid drug clearance through the Eustachian tube (half-life of corticosteroids ≈27 min) [16,140]. Recent advancements employ hydrogel-based formulations and gelatin sponges to prolong drug retention at the RWM interface [77,141].

Intracochlear administration aims to inject drugs directly into the lymphatic fluid, providing a more precise approach compared to intratympanic delivery, with better control over dosage gradients and reduced systemic toxicity [142]. However, this technique is surgically demanding, carries a high risk of trauma, and is primarily used in animal experiments, with limited clinical applications, particularly in gene therapy [143]. Emerging solutions include osmotic micropumps and reciprocating perfusion systems that enable sustained, low-flow drug delivery while minimizing mechanical trauma to cochlear structures [16,138].

While local delivery methods can bypass the BLB, they fail to achieve true non-invasiveness due to inherent procedural limitations. The relatively mature intratympanic administration still requires tympanic membrane penetration using needles or preexisting perforations, which introduces risks of complications including otalgia, vertigo, and persistent eardrum defects [137]. Furthermore, this method suffers from inconsistent drug diffusion across the round window membrane (RWM) and variable retention rates in the middle ear cavity, leading to unreliable dosage precision and strong basoapical concentration gradients in the cochlea [16,84]. The more direct intracochlear injection approach faces even greater clinical barriers due to its invasive nature that the procedure demands specialized surgical skills to access the cochlea through RWM perforation or cochleostomy, with risks of damaging the fragile sensory epithelium through perilymph volume or pressure changes [138].

Therefore, researchers are also exploring the intrinsic properties of the BLB itself, seeking to develop drugs and drug carriers that can penetrate the BLB following systemic administration.

## 9. Innovative Approaches to Penetrate the BLB

**Figure 3:**
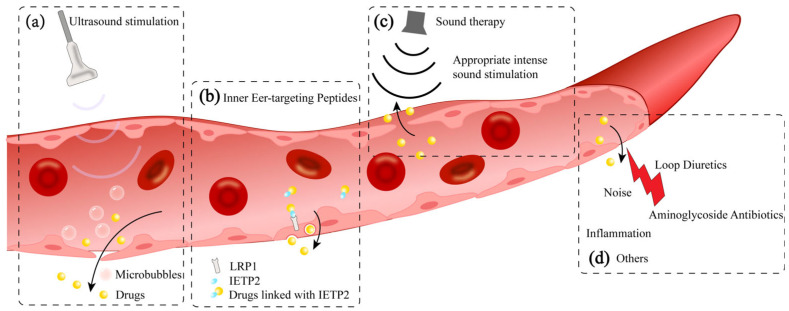
New methods to penetrate blood-labyrinth barrier through its intrinsic nature: (**a**) Ultrasound stimulation: combining the use of low-pressure pulsed ultrasound and microbubbles can temporarily open the BLB, allowing for the effective delivery of intravenous drugs into the cochlea. (**b**) Inner ear-targeting peptides: conjugated with inner ear-targeting peptide 2 (IETP2), drugs can transport into blood-labyrinth barrier via low-density lipoprotein receptor-related protein 1 (LRP1). (**c**) Sound therapy: appropriate intense sound stimulation (without causing permanent damage to cochlea) can temporarily increase the permeability of the BLB and promote the paracellular entry route of drugs into the cochlea. (**d**) Others: under certain circumstances, such as noise exposure, ototoxic drugs, and inflammation, the structure of the BLB can be damaged and its permeability increased, creating an opportunity to transport drugs through systemic injection that would initially be unable to pass through.

Under certain circumstances, the permeability of the BLB changes, creating an opportunity to transport drugs through systemic injection that would initially be unable to pass through (Figure 3). Proteins expressed on the surface of BLB cells may also serve as potential targets for therapeutic drug delivery routes. For instance, ototoxic aminoglycosides can cross the BLB through marginal cells, suggesting that finding other drug carriers with similar properties might be applicable in drug development [90].

### 9.1. Drug-Induced Permeability Change of BLB

The most classic circumstance is the aminoglycoside antibiotics. Aminoglycoside antibiotics reach peak concentrations in inner ear fluids much more slowly than those in the blood, leading to two completely different outcomes regarding the combined use of aminoglycoside antibiotics and loop diuretics. When gentamicin concentration in the blood is higher than in the lymphatic fluid, using ethacrynic acid to disrupt the BLB can accelerate the entry of ototoxic drugs into the cochlea, rapidly reaching concentrations that damage hair cells [109]. Conversely, when the gentamicin concentration in the blood is lower than in the lymphatic fluid, using ethacrynic acid to disrupt the BLB helps promote the excretion of ototoxic drugs from the inner ear fluid, thereby reducing their continuous damage to hair cells [109]. A similar strategy can be used in other drugs that initially could not penetrate the blood-labyrinth barrier. The strategic administration of loop diuretics (e.g., ethacrynic acid) can transiently enhance blood-brain barrier permeability. This pharmacological modulation creates a therapeutic window for improved penetration of antibiotics or anti-inflammatory medications into protected anatomical compartments.

### 9.2. Ultrasound Therapy Assisted by Microbubbles

An innovative therapeutic approach using sound, specifically low-pressure pulsed ultrasound assisted by microbubbles (USMB), aims to open the BLB [144]. When microbubbles are combined with ultrasound, the expression of tight junction proteins like ZO-1 and occludin is reduced, allowing for the effective delivery of intravenous drugs such as hydrophilic dexamethasone sodium phosphate (DSP) into the cochlea [144] (Figure 3a). This method has been used and clinically assessed in research related to the BBB [33,145], but its application to the BLB is still in the initial stage (Figure 3a).

### 9.3. Inner Ear-Targeting Peptides

In the BBB, LRP1 targeting has been achieved by encapsulating IgG antibodies in polymers modified with the peptide Angiopep-2 and delivering them to the central nervous system of mice [146]. Although it is unclear whether the BLB can transport drugs via vesicular transport, low-density lipoprotein receptor-related protein 1 (LRP1), which is highly expressed at the apex of the stria vascularis and is similar in structure to the BBB’s LRP1, indicates the potential for macromolecules to cross the BLB through vesicular transport. This approach has been applied to drug development targeting the BLB. Researchers have synthesized LRP1-specific binding ligands—inner ear-targeting peptide 2 (IETP2)—and linked small molecular compounds to these peptides to achieve targeted drug delivery to the inner ear [20]. These compounds conjugated with IETP2 were successfully transported into the cochlea, demonstrating the potential of this drug delivery strategy (Figure 3b). The successful application of this peptide modification strategy in the blood-brain barrier (BBB) and blood-labyrinth barrier (BLB) may be extended to various therapeutic agents, enabling targeted drug delivery to the inner ear through direct intravenous injection.

### 9.4. Sound Therapy

In specific circumstances, mild stimulation of the cochlea does not cause permanent damage; instead, it may enhance the cochlea’s resistance to stronger stimuli that could lead to permanent damage [147]. Exposing the cochlea to a sound that is non-damaging activates the ear’s protective mechanisms and increases its resilience to potential future permanent injuries [148]. Sound conditioning therapy is proposed to increase BLB permeability under certain noise exposure conditions, which makes it easier for drugs to enter the cochlea through the paracellular pathway [149]. Specifically, high-intensity noise exposure can disrupt the structural integrity of the BLB, but under conditions of sustained sound stimulation at higher intensities without causing permanent damage (90 dB SPL, 8–16 kHz, 2 h), it can controllably and temporarily (within 6 h) increase the permeability of the BLB, promoting the paracellular entry of blood-borne drugs into the cochlea [149] (Figure 3c). In fact, after this sound conditioning, dexamethasone phosphate (DEX-P), a drug that is transported by paracellular pathways, has been shown to have a higher concentration in the cochlea. This provides a viable non-invasive adjunctive approach for inner ear treatment.

### 9.5. Route of Cerebrospinal Fluid Conduit

Given the connection between the cerebrospinal and inner ear fluids via the cochlear aqueduct, administration of drugs through the cerebrospinal fluid is feasible [17]. The cochlear aqueduct serves as a critical anatomical conduit between the inner ear and the CSF compartment. Structurally, it connects the scala tympani of the cochlea to the subarachnoid space in the posterior cranial fossa via a funnel-shaped medial aperture, mitigating inner ear exposure to CSF pressure fluctuations in the posterior fossa [17,150]. This mechanism is thought to protect delicate cochlear structures from abrupt pressure changes, though its efficacy in adults is limited by anatomical narrowing.

The intracisternal injection of the Slc17A8 gene via an adeno-associated virus (AAV) has successfully restored hearing in deaf Slc17A8 knockout mice [151]. However, this delivery strategy requires highly specialized carriers to meet the unique anatomical challenges of inner ear tissues. This has driven the development of targeted vectors like AAV2.8 and AAV-i.e., engineered specifically for precision delivery in the central nervous system, including cochlear [141].

### 9.6. Inflammation-Driven Delivery

Inflammatory conditions of the inner ear may paradoxically improve drug delivery efficiency. During local inner ear inflammation, increased BLB permeability and upregulated transporters facilitate systemic drug accumulation in inner ear tissues [104,152]. In such cases, systemic drug administration may become more effective for targeting inner ear organs. Exploring the mechanisms of BLB opening during inflammation could provide new insights for drug development (Figure 3d).

While appearing innocuous, these innovative approaches present distinct advantages and potential limitations (Table 3). Comprehensive studies are required to investigate the feasibility of implementing these concepts. However, even if drugs can be non-invasively delivered to the inner ear, achieving an effective distribution of their concentration within the inner ear remains highly challenging. After local drug administration, a gradient of drug concentration from the cochlear base to the apex can be detected, limiting the potential therapeutic effect on the cochlear apex [136]. This phenomenon is also likely to occur when drugs are systemically delivered to the inner ear. In addition, the strength of the BLB may not be consistent across individuals. For those with existing inner ear diseases, their BLB may be more easily opened. Therefore, controlling the intensity of the method used to open the BLB (such as sound or ultrasound) is also a major challenge in research.

**Table 3:** Innovative approaches to penetrate the blood-labyrinth barrier.

Method	Mechanism	Advantages	Potential Limitations
Drug-induced BLB permeability changes [109]	Combined use of ototoxic drugs with diuretics to disrupt BLB integrity	Controllable time window; bidirectional drug concentration modulation	Risk of ototoxicity aggravation; difficulty in precise timing
Microbubble-assisted ultrasound therapy [144]	Low-pressure pulsed ultrasound + microbubble cavitation to transiently open tight junctions	Non-invasive; enables large-molecule delivery; spatiotemporal precision	Potential local inflammation; long-term safety requires validation
Inner ear-targeting peptide [20]	LRP1 receptor-mediated transcytosis targeting stria vascularis marginal cells	High specificity; avoids systemic side effects (small molecules)	Limited by receptor expression; low macromolecule delivery efficiency
Sound therapy [149]	Sound conditioning strategy temporarily increases paracellular permeability	Fully non-invasive; synergizes with cochlear protective mechanisms	Requires personalized acoustic parameters; potential sound trauma
Cerebrospinal fluid conduit route [151]	Intrathecal injection via cochlear aqueduct (AAV-mediated gene delivery)	Bypasses BLB; ideal for genetic hearing loss	Low efficiency due to fluid dynamics; CNS side effects
Inflammation-driven delivery [70,106,108]	Systemic drug administration during BLB permeability elevation in pathological states	Pathophysiology-synchronized targeting; dual therapeutic effects	Intervention timing dependent on disease progression; may worsen inflammation

## 10. Strategies to Protect and Regulate Blood-Labyrinth Barrier Function

Up until now, effective drugs specifically targeting the dysfunction of the BLB have not been developed yet. However, the normal function of the BLB needs sufficient blood flow to support the high metabolism of the stria vascularis.

### 10.1. Gene Therapy

Due to the components of the BLB being rather complex, gene therapy targeting the entire BLB is quite challenging. However, there are gene therapy strategies available for specific cells of the BLB. Vascular endothelial growth factor-A165 (VEGFA165) gene therapy shows promise in restoring stria vascularis PCs, improving blood supply, and reducing hearing loss in noise-exposed animals [153]. After transfecting VEGFA165 into PCs using AAV and subsequently transplanting these PCs into the cochlea following acoustic trauma, both blood flow and PCs proliferation can be enhanced [101]. In the JCR syndrome mouse model, Kcnq1 gene therapy effectively restores Kcnq1 expression in marginal cells and rescues the function of the stria vascularis [119].

### 10.2. Rescue the Injured Cells of BLB

Cochlear fibrocytes in the lateral wall are essential for potassium ion recycling to the stria vascularis; hence, the degeneration or injury of these fibrocytes may result in hearing loss [154]. Since fibrocytes are believed to originate from mesenchymal cells, transplanting mesenchymal stem cells into the cochlea has the potential to differentiate into fibrocytes. Indeed, the transplantation of isolated bone-marrow-derived stem cells or hematopoietic stem cells into the cochlea has resulted in the expression of marker proteins characteristic of ion-transporting fibrocytes, such as cx26 and Na^+^-K^+^-ATPase, and improved vascular integrity [155]. Transplanted MSCs, detected in the spiral ligament, adopt fibrocyte-like morphologies and restore potassium recycling pathways, accelerating hearing recovery in fibrocyte-dysfunction models [156]. After transplantation, the transplanted MSCs are detected in the spiral ligament area and express marker proteins specific to fibrocytes. PCs are also one target of cell transplantation therapy since they play roles in regulating blood flow and vascular permeability. Post-acoustic trauma, transplantation of neonatal cochlear PCs reduces strial vascular atrophy by promoting angiogenesis and blood flow, likely through VEGF-A signaling, thereby preserving EP and auditory function [101]. These strategies highlight the regenerative potential of stem or progenitor cells in reversing fibrocyte-associated cochlear pathology.

## 11. Limitations and Future Perspectives

### 11.1. Limitations

This review on the BLB and inner ear drug delivery strategies, while comprehensive, exhibits several limitations. Primarily, the reliance on preclinical animal studies and in vitro models limits direct clinical translatability, as human-specific BLB dynamics and therapeutic responses remain underexplored. On the other hand, since we intend to introduce some newly developed non-invasive inner ear drug delivery strategies, the approaches we describe in this review could potentially omit other conflicting evidence or recent advancements in BLB modulation, such as emerging nanoparticle technologies or CRISPR-based therapies.

Additionally, while innovative strategies like ultrasound-mediated delivery and gene therapy are highlighted, the table does not sufficiently address critical safety concerns, long-term efficacy, and scalability challenges for clinical adoption due to a lack of relevant research. The review also does not systematically compare the efficacy, risks, or technical feasibility of the discussed methods, leaving gaps in practical guidance for future researchers. These limitations necessitate future research focusing on integrating human data, utilizing advanced microscopy and imaging techniques, and uncovering in-depth molecular mechanisms underlying the BLB, thereby truly translating these new strategies into clinical use.

### 11.2. Future Pespectives

Recent progress in imaging and modeling techniques has significantly deepened our understanding of BLB structure and function. Studies have uncovered critical aspects of ion homeostasis, barrier permeability, and the role of various cell types within the BLB, yet its full anatomical structure and the interactions among its various cell types remain unclear. Future research, using advanced microscopy and imaging techniques, could provide a more detailed view of the BLB’s structure, offering a deeper understanding of its complexity and variability and establishing a foundation for further studies.

The in-depth study of the molecular mechanisms of the BLB is also one of the key directions for the future. The function of the BLB depends on the coordinated action of various ion channels and transport proteins. Understanding these molecular mechanisms will not only help reveal the specific role of the BLB in maintaining inner ear homeostasis but will also provide a theoretical basis for developing therapeutic strategies for inner ear diseases.

The BLB poses significant challenges for targeted drug delivery to the inner ear. Emerging evidence reveals the BLB’s dynamic responsiveness to external stimuli—including acoustic trauma, inflammatory mediators, and ototoxic substances—highlighting both its remarkable adaptability and inherent vulnerability. Despite these insights, developing non-invasive or minimally invasive techniques capable of achieving efficient and selective inner ear targeting continues to present formidable scientific and technical hurdles.

Notably, some newly developed non-invasive inner ear drug delivery strategies have presented promising therapeutic potential. These include sound therapy, ultrasound therapy assisted by microbubbles, inner ear-targeting peptides, and the administration route via the cerebrospinal fluid conduit. Other strategies, such as lipid-soluble nanoparticles, receptor-based approaches, and the use of transporters or vesicles as natural delivery pathways, have also shown promise for more efficient and less invasive drug delivery. These emerging strategies collectively aim to enhance delivery efficiency while reducing invasiveness, thereby optimizing therapeutic efficacy and minimizing systemic adverse effects.

## Abbreviations

The following abbreviations are used in this manuscript:

BLB	Blood-labyrinth barrier
BM	Basement membrane
CSF	Cerebrospinal fluid
HRP	Horseradish peroxidase
EP	Endocochlear potential
SV	Stria vascularis
PCs	Pericytes
PVM/Ms	Perivascular macrophage-like melanocytes
ECs	Endothelial cells
MCs	Marginal cells
ICs	Intermediate cells
BCs	Basal cells
TMPA	Trimethylphenylammonium
BBB	Blood-brain barrier
BRB	Blood-retinal barrier
LRP1	Low-density lipoprotein receptor-related protein 1
MSCs	Mesenchymal stem cells
AAV	Adeno-associated virus
IETP2	Inner ear-targeting peptide 2
VEGF	Vascular endothelial growth factor
TNF-α	Tumor necrosis factor-alpha
HIF-1α	Hypoxia-inducible factor-1α
PEI	Polyethyleneimine
TRPA1	Transient receptor potential channels
USMB	Ultrasound assisted by microbubbles
DSP	Hydrophilic dexamethasone sodium phosphate
DEX-P	Dexamethasone phosphate
